# Nomogram model based on clinical factors and autonomic nervous system activity for predicting residual renal function decline in patients undergoing peritoneal dialysis

**DOI:** 10.3389/fnins.2024.1429949

**Published:** 2024-11-01

**Authors:** Jing Wang, Zhenye Chen, Yaoyu Huang, Yujun Qian, Hongqing Cui, Li Zhang, Yike Zhang, Ningning Wang, Hongwu Chen, Haibin Ren, Huijuan Mao

**Affiliations:** ^1^Department of Nephrology, The First Affiliated Hospital of Nanjing Medical University, Jiangsu Province Hospital, Nanjing, China; ^2^Department of Cardiology, The First Affiliated Hospital of Nanjing Medical University, Jiangsu Province Hospital, Nanjing, China

**Keywords:** chronic kidney disease, heart rate variability, skin sympathetic nerve activity, peritoneal dialysis, nomogram model

## Abstract

**Background:**

Several heart rate variability (HRV) parameters were reported to be associated with residual renal function (RRF) in patients undergoing continuous ambulatory peritoneal dialysis (CAPD). However, it is unclear whether using HRV or other autonomic nervous system (ANS) activity indexes can predict RRF decline in CAPD patients.

**Methods:**

Patients undergoing CAPD in 2022 from the First Affiliated Hospital of Nanjing Medical University were enrolled in this study. Their clinical characteristics, 5-min HRV parameters and average voltage of 5-min skin sympathetic nerve activity (aSKNA) were collected. According to the 12-month glomerular filtration rate (GFR) decline rate compared with the upper quartile, these patients were categorized into two groups: RRF decline (RRF-D) group and RRF stable (RRF-S) group. Clinical factors and ANS activity indexes for predicting 1-year RRF decline were analyzed using logistic regression, and a nomogram model was further established. The relationships between volume load related indexes and aSKNA were displayed by Spearman's correlation graphs.

**Results:**

Ninety-eight patients (53 women, average age of 46.7 ± 13.0 years old) with a median dialysis vintage of 24.5 months were enrolled in this study. Seventy-three patients were categorized into the RRF-S group and 25 patients into the RRF-D group. Compared with RRF-S group, patients in the RRF-D group had higher systolic blood pressure (BP; *p* = 0.019), higher GFR (*p* = 0.016), higher serum phosphorous level (*p* = 0.030), lower total Kt/V (*p* = 0.001), and lower levels of hemoglobin (*p* = 0.007) and albumin (*p* = 0.010). The RRF-D group generally exhibited lower HRV parameters and aSKNA compared with the RRF-S group. A nomogram model included clinical factors (sex, systolic BP, hemoglobin, GFR, and total Kt/V) and aSKNA showed the largest AUC of 0.940 (95% CI: 0.890–0.990) for predicting 1-year RRF decline.

**Conclusion:**

The nomogram model included clinical factors (sex, systolic BP, hemoglobin, GFR and total Kt/V) and ANS activity index (aSKNA) might be a promising tool for predicting 1-year RRF decline in CAPD patients.

## Introduction

Peritoneal dialysis (PD) accounts for ~11% of all patients undergoing dialysis for treatment of end-stage kidney disease (ESKD; Teitelbaum, 2021). Recent studies have demonstrated that the maintenance of residual renal function (RRF) is independently associated with increased survival in patients on PD (Chen et al., 2020), while rapid decline in RRF indicates a high risk of mortality in patients undergoing PD, especially in the first year (Wang et al., 2019). One research in Taiwan indicates that the median rate of decline in RRF is 0.89 per year, and the average time progressing to anuria is 30 months (Liao et al., 2009). Therefore, the preservation of RRF is an important therapeutic strategy in the management of PD patients.

Accumulated evidence suggests that prevalence and severity of autonomic nervous system (ANS) dysfunction increases as CKD progresses toward ESKD (Thapa et al., 2010). Heart rate variability (HRV) is a traditional non-invasive method to assess autonomic function, while skin sympathetic nerve activity (SKNA) is a newly proposed method to reflect cardiac sympathetic nerve activity, which has been shown in animal experiments to be linearly correlated with the direct recording of stellate ganglion nerve activity (Jiang et al., 2015). Although several HRV parameters were reported to be associated with RRF in patients undergoing continuous ambulatory peritoneal dialysis (CAPD; Tang et al., 2012), it is unclear whether using ANS activity indexes can predict RRF decline. In this study, we assessed the predictive value of a nomogram model based on ANS activity indexes and clinical factors for RRF decline in CAPD patients.

## Materials and methods

### Study population

Ninety-eight patients who underwent CAPD were enrolled at the First Affiliated Hospital of Nanjing Medical University between January and December 2022. All participants were aged 18–75 years and had been receiving CAPD treatment for at least 6 months. The exclusion criteria were as follows: (1) with urine output < 100 ml for 6 months; (2) history of kidney transplantation; (3) fasting blood glucose on the day of evaluation ≥200 mg/dL; (4) presence of fever, infection, pregnancy or lactating women; (5) severe congenital heart disease or ventricular arrhythmia; (6) episodes of acute myocardial infarction, stroke, or a major surgical procedure within the past 3 months.

### Baseline characteristics

Baseline characteristics of the patients were collected as follows: demographic information, comorbidities, causes of ESKD and history of CKD treatment.

### Measurements of blood parameters and blood pressure

Venous blood samples were collected in the morning before dialysis after overnight fasting, and were tested for routine blood, creatinine, urea, uric acid, albumin, total cholesterol, triglyceride, β2-microglobulin, serum calcium, serum chlorine, serum phosphorus, alkaline phosphatase, intact parathyroid hormone (iPTH), and pro-BNP. Systolic and diastolic blood pressure (BP) were measured just before the exchange of morning dialysate.

### RRF and patient classification

RRF was measured as glomerular filtration rate (GFR) using the mean of urea and creatinine clearances (van Olden et al., 1996). In this study, patients' GFR at 12 months during follow-up were reviewed for GFR decline rate, which was calculated as (GFR at 12 months—GFR at baseline)/12. According to the status of RRF, patients were categorized into two groups: RRF decline (RRF-D) group, with GFR decline rate higher than the upper quartile of the study population, and RRF stable (RRF-S) group, with GFR decline rate lower than the upper quartile of the study population.

### Single-lead ECG and SKNA recording

Each patient underwent a 5-min recording before the exchange of morning dialysate using a custom-made device which can record single-lead ECG and SKNA simultaneously (Xing et al., 2022; Zhang et al., 2022). The device had three electrodes (3M™ Red Dot Monitoring Electrode, #2570) placed in specific locations (the left subclavian, the right subclavian and the right abd omen) and recorded signals continually, with a sampling rate of 4,000 Hz. The patients were instructed to stay supine and avoid unnecessary movement during the recording. Electronic instrument usage, which could produce signal artifacts, were avoided during recordings.

### HRV analysis

The 5-min HRV analysis was based on the PhysioNet Cardiovascular Signal Toolbox by Vest et al. (2018). Beat-to-beat RR intervals were extracted and underwent time-domain analysis, frequency domain analysis and non-linear analysis. Lomb–Scargle periodogram was used as the default method for frequency analysis.

The time-domain analysis includes the mean interval of normal sinus beats (NNmean), standard deviation of all sinus RR intervals (SDNN), square root of the mean square of differences between adjacent normal-to-normal intervals (RMSSD), percentage of the number of pairs of adjacent normal-to-normal intervals differing >50 ms in the total normal-to-normal intervals (pNN50) and deceleration capacity (DC). The frequency-domain analysis consists of total power (TP), high-frequency power (HF), ultra-low frequency power (ULF), very low-frequency power (VLF), low-frequency power (LF) and the ratio of low to high-frequency power (LF/HF). The non-linear analysis consists of SD1, SD2, SD1/SD2, sample entropy (SampEn) and approximate entropy (ApEn).

### SKNA data processing

**Figure 1:**
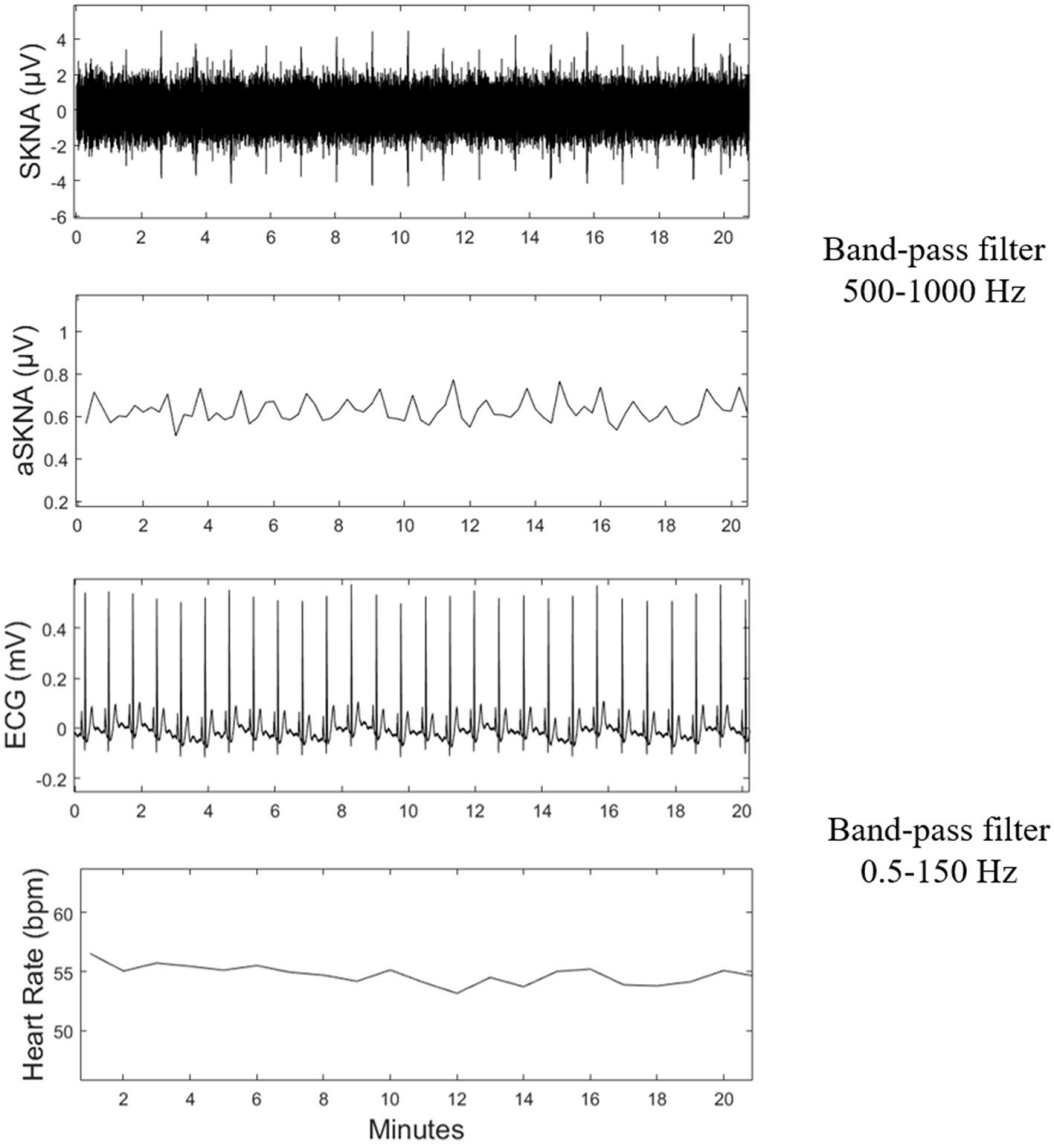
Schematic of physical signal data processing. SKNA, skin sympathetic nerve activity; aSKNA, average voltage of 5-min SKNA.

SKNA was derived from the raw data after bandpass filtering at 500–1,000 Hz (Kusayama et al., 2020). Next, third quartiles were calculated as Q1, Q2, Q3, respectively. The deviation of Q1 and Q3 were defined as interquartile range (IQR). Mild outliers, the data below Q1-1.5^*^IQR or above Q3+1.5^*^IQR, were excluded. Then the average voltage of SKNA (aSKNA) was derived from the average of the 5-min time period. The representative signals processed from the raw data are shown in Figure 1.

### Measurement of dialysis adequacy and volume status

Small solute removal was determined by measurement of total (PD and renal) weekly urea Kt/V using standard methods (Gotch and Sargent, 1985). The contributions to total Kt/V (tKt/V) by PD (pKt/V) and RRF (rKt/V) were estimated separately (Auguste and Bargman, 2023). The volume of urea distribution (V) was derived using Watson's formula (Watson et al., 1980).

Assessment of volume status was performed on a body composition monitor (BCM; Fresenius Medical Care, Bad Homburg, Germany) by the same experienced nurse according to the instrument instructions. The patient's clinical parameters containing age, gender, height, and weight were inputted into the device. Electrodes were placed on the hand and foot of patients' non-dominant side, and then electrical responses were collected every 50 discrete frequencies from 5 to 1,000 kHz. Given the measured impedance information, overhydration (OH), total body water (TBW), extracellular water (ECW) and intracellular water (ICW) were calculated by the equations proposed by Moissl et al. (2006).

### Statistical analysis

Distributed data were reported as mean ± standard deviation, the *t*-test and non-parametric test were utilized to compare continuous variables between groups. Categorized variables were presented as frequency and analyzed by Chi-square test. Baseline characteristics were compared using the *t*-test or Mann-Whitney *U* test for continuous variables depending on the data distribution, and chi-square test was used for categorical variables.

Binary logistic regression was used for univariate and multivariate analyses to explore the independent risk factors of RRF decline. In the multivariate analysis, the indicators with *p* < 0.10 in the univariate analysis and the basic factors including age, sex, and dialysis vintage were included. Spearman's correlation analyzed the relationships between HRV parameters, aSKNA and other clinical data. A nomogram model for predicting 1-year RRF decline of these patients was developed based on multivariable logistic regression analysis results. The accuracy and discrimination of the nomogram model were evaluated by the area under the curve (AUC) value of the receiver operating characteristic (ROC) curve using the “timeROC” package in R.

All the statistical analyses were performed using R Software Version 3.6.2 (The R Foundation for Statistical Computing), and two-sided *p* < 0.05 was considered statistically significant unless stated otherwise.

## Results

### Baseline characteristics

**Figure 2:**
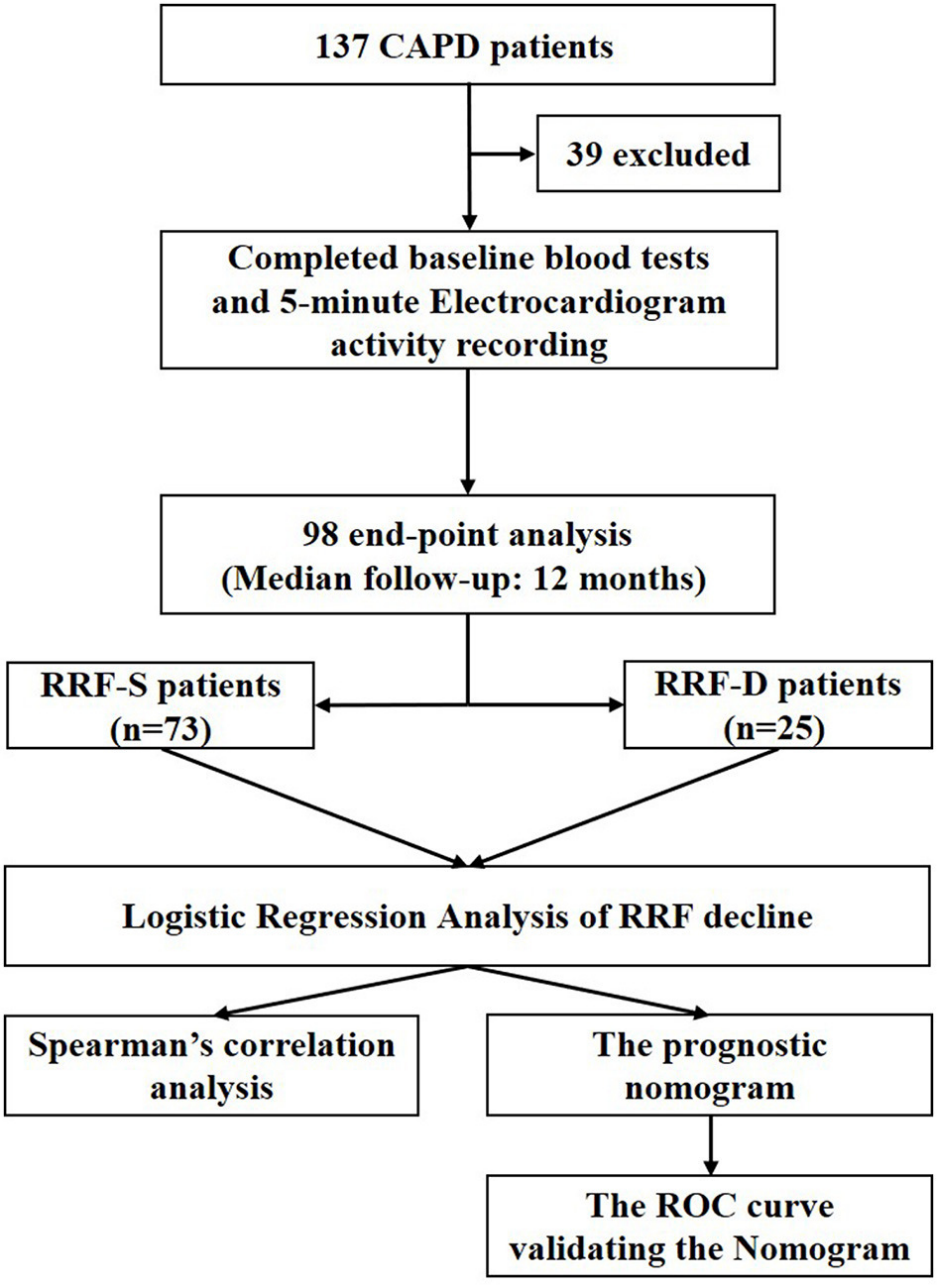
Flow chart of the study design. CAPD, continuous ambulatory peritoneal dialysis; RRF, residual renal function; RRF-S, residual renal function stable group; RRF-D, residual renal function decline group.

A total of 98 CAPD patients (53 women, average age 46.7 ± 13.0 years old with a median dialysis vintage of 24.5 months) were enrolled in this study and were categorized into two groups: 73 patients in the RRF-S group and 25 patients in the RRF-D group. The flowchart of study was shown in Figure 2. Baseline characteristics are summarized in Table 1. There were no significant differences in the baseline clinical characteristics between the two groups (Table 1), except patients in RRF-D group had lower total Kt/V, higher systolic BP, ECW and baseline RRF, lower levels of hemoglobin and albumin, and higher levels of serum phosphorous (all *p* < 0.05).

**Table 1:** Baseline characteristics of patients undergoing CAPD.

	**Overall (*n* = 98)**	**RRF-S (*n* = 73)**	**RRF-D (*n* = 25)**	***P*-value**
**Clinical characteristics**				
Male, *n* (%)	45 (45.9)	31 (42.5)	14 (56.0)	0.256
Age (years)	46.7 ± 13.0	45.4 ± 12.7	50.3 ± 13.1	0.102
BMI (kg/m^2^)	21.6 (19.9, 24.1)	21.6 (19.9, 23.6)	21.9 (19.8, 25.1)	0.496
Systolic BP (mmHg)	136.9 ± 21.0	134 ± 18.8	145.5 ± 25.0	0.019
Diastolic BP (mmHg)	89.1 ± 13.6	88 ± 13.7	92.3 ± 13.0	0.184
Diabetic mellitus	2 (2.0)	1 (1.4)	1 (4.0)	0.447
Dialysis vintage (months)	24.5 (12.0, 45.3)	25 (13, 45.5)	16 (9, 45)	0.309
Previous peritonitis, *n* (%)	4 (4.1)	2 (2.7)	2 (8.0)	0.251
**Medication history**, ***n*** **(%)**				
Use of Dihydropyridine CCBs	68 (69.4)	52 (71.2)	16 (64.0)	0.616
Use of ACEI/ARB	58 (59.2)	41 (56.2)	17 (68.0)	0.352
Use of β-blocker	58 (59.2)	44 (54.8)	14 (56.0)	0.815
**Baseline laboratory findings**				
Hemoglobin (g/L)	110.5 ± 17.1	113.2 ± 15.9	102.5 ± 18.5	0.007
Total cholesterol (mmol/L)	4.5 ± 1.2	4.5 ± 1.2	4.4 ± 1.2	0.764
Triglyceride (mmol/L)	1.7 (1.2, 2.3)	1.6 (1.3, 2.3)	1.7 (0.9, 2.0)	0.473
Albumin (g/L)	38.4 ± 4.0	39 ± 3.6	36.7 ± 4.5	0.010
CRP (mg/L)	2.9 (1.4, 4.8)	2.6 (1.3, 4.5)	3.5 (1.5, 7.5)	0.104
β2-microglobulin (mg/L)	35.1 (22.2, 42.4)	35.4 (22, 42.6)	30.0 (22.7, 41.1)	0.725
Uric acid (mmol/L)	372.5 (303.8, 434.0)	370 (287, 434)	375.0 (318.0, 468.5)	0.383
Adjust serum calcium (mmol/L)	2.4 ± 0.2	2.4 ± 0.1	2.3 ± 0.2	0.058
Serum chlorine (mmol/L)	100.3 ± 4.3	100 ± 4.2	101.2 ± 4.6	0.197
Serum phosphorus (mmol/L)	1.7 (1.4, 2.1)	1.6 (1.4, 2)	2.0 (1.6, 2.4)	0.030
ALP (U/L)	71 (58.8, 91.8)	72.5 (59.0, 94.8)	66.0 (56.8, 86.5)	0.406
iPTH (pg/mL)	207.3 (111, 322.4)	195.4 (103.7, 297.9)	257.3 (142.3, 387.1)	0.090
Pro-BNP (ng/L)	1,470.5 (550.4, 3,804.5)	1,406 (579.1, 3,020.5)	2,738 (444.8, 9,374)	0.108
GFR (mL/min)	31.1 (10.2, 53.3)	24.9 (8.1, 53.1)	43.4 (24.6, 70.0)	0.016
Total Kt/V	2.0 ± 0.5	2.1 ± 0.4	1.7 ± 0.4	0.001
**Echocardiography**				
LVEF (%)	63 (61.9, 64.6)	63.0 (61.9, 64.7)	62.7 (61.9, 63.7)	0.317
LVDd (mm)	47 (44, 49)	47 (44, 49)	46 (43, 49)	0.895
E/A	0.8 (0.7, 1.0)	0.8 (0.7, 1.2)	0.8 (0.6, 0.9)	0.079
E/e'	8.4 (7.1, 9.5)	8.5 (7.0, 9.6)	8.3 (7.3, 9.3)	0.819
**Bioelectrical impedance**				
OH	1.4 (0.7, 2.4)	1.4 (0.6, 2.1)	2.3 (0.8, 4.1)	0.080
TBW	35.3 ± 7.2	34.8 ± 7.2	36.8 ± 7.3	0.309
ECW	15.9 ± 3.0	15.5 ± 3.1	16.9 ± 2.6	0.084
ICW	18.4 (15.7, 23.2)	18.0 (15.7, 23.1)	18.4 (15.8, 24.4)	0.484

CAPD, continuous ambulatory peritoneal dialysis; RRF-S, residual renal function stable group; RRF-D, residual renal function decline group; BMI, body mass index; BP, blood pressure; CCB, calcium channel blocker; ACEI/ARB, angiotensin-converting enzyme inhibitors/angiotensin receptor blocker; GFR, glomerular filtration rate; ALP, alkaline phosphatase; iPTH, intact parathyroid hormone; pro-BNP, pro-brain natriuretic peptide; LVEF, left ventricular ejection fraction; LVDd, left ventricular end diastolic dimension; E/A, ratio of early transmitral flow velocity (E) and late diastolic mitral flow velocity (A); E/e', ratio of early transmitral flow velocity (E) and mitral annular velocity during diastole (e'); OH, overhydration; TBW, total body water; ECW, extracellular water; ICW, intracellular water.

Data are presented as mean ± SD, numbers and percentages, as appropriate.

### Baseline HRV parameters and aSKNA

The RRF decline group generally exhibited lower HRV parameters and aSKNA values compared with the RRF stable group, except LF/HF. The differences in SDNN, TP, ULF, VLF, LF, HF, DC, SD2, ApEn, and aSKNA were statistically significant (*p* < 0.05), while there was no significant difference in other HRV parameters between two groups (Table 2).

**Table 2:** Baseline HRV parameters and aSKNA in patients undergoing CAPD.

**ANS activity indexes**	**Overall (*n* = 98)**	**RRF-S (*n* = 73)**	**RRF-D (*n* = 25)**	***P*-value**
**HRV parameters**				
**Time domain**				
NNmean (ms)	784.2 ± 112.9	802.5 ± 120.8	732.7 ± 116.2	0.018
SDNN (ms)	34.5 (22.6, 42.8)	38.3 (24.1, 47.7)	26.3 (18.6, 35.9)	0.010
RMSSD (ms)	16.8 (11.9, 35)	18.9 (11.9, 36.8)	14.7 (10.4, 30.3)	0.345
pNN50 (%)	0.01 (0, 0.04)	0.01 (0, 0.04)	0 (0, 0.03)	0.148
DC	4.7 (3.1, 6)	5.6 (3.5, 6.3)	3.9 (2, 4.6)	0.008
**Frequency domain**				
ULF	86.8 (32.9, 518.1)	114.5 (38.9, 670)	43.8 (22.6, 112.2)	0.01
VLF	574.5 (293.4, 1,227)	775.7 (775.7, 1,227)	397.8 (201.7, 574.5)	0.001
LF	295.1 (114, 453.6)	430 (162, 602.4)	210.5 (60.6, 327.1)	0.007
HF	147.9 (68.3, 295.3)	147.9 (101.1, 355.6)	76.1 (30.3, 261.5)	0.004
LF/HF	1.7 (0.9, 2.9)	1.6 (0.9, 2.9)	2.1 (0.8, 3.9)	0.411
TP	1,316.8 (567.5, 2,475)	2,070.6 (618.6, 2,479)	800.4 (456.8, 1,197.7)	0.001
**Non-linear**				
SD1	12.5 (8.4, 24.8)	13.4 (13.4, 26)	10.4 (7.3, 21.4)	0.305
SD2	43.1 (28.5, 53.6)	50.9 (30.7, 58.4)	33.9 (23.9, 41.9)	0.001
SD1/SD2	0.3 (0.2, 0.6)	0.3 (0.2, 0.5)	0.4 (0.2, 0.6)	0.286
SampEn	1.4 (1.1, 1.7)	1.4 (1.1, 1.6)	1.5 (1.2, 1.8)	0.065
ApEn	1.1 (1, 1.2)	1.1 (1, 1.1)	1.1 (1, 1.2)	0.049
**SKNA**				
aSKNA (μV)	1.3 (1, 1.4)	1.3 (1.1, 1.5)	1.1 (0.9, 1.1)	< 0.001

CAPD, continuous ambulatory peritoneal dialysis; HRV, heart rate variability; NNmean, mean interval of normal sinus beats; SDNN, standard deviation of normal-to-normal R-R intervals; RMSSD, root mean square of differences between adjacent normal R–R intervals; pNN50, proportion of adjacent R-R intervals differing by 50 ms over 24 h; ULF, ultral low frequency power; VLF, very low frequency power; LF, low frequency power; HF, high frequency power; LF/HF, ratio of LF power to HF power; TP, total power; DC, deceleration capacity; SampEn, sample entropy; ApEn, approximate entropy; SKNA, skin sympathetic nerve activity; aSKNA, average voltage of 5-min skin sympathetic nerve activity.

Data are presented as mean ± SD, numbers and percentages, as appropriate.

### Clinical factors and ANS activity indexes for predicting RRF decline

Univariate analysis showed that clinical characteristics including systolic BP, GFR, hemoglobin, albumin, phosphorus and total Kt/V, ANS indexes including NNmean, SDNN, ULF, VLF, LF, TP, DC, SD2, and aSKNA were associated with RRF decline (Table 3).

**Table 3:** Univariate and multivariable logistic regression analysis for predicting RRF decline.

	**Univariate logistic regression analysis**		**Multivariate logistic regression analysis**	
**RRF-D**	**OR (95% CI)**	***P*** **-value**	**OR (95% CI)**	***P*** **-value**
Age	1.031 (0.994, 1.070)	0.105		
Female sex	1.724 (0.690, 4.309)	0.244	7.468 (1.054, 52.942)	0.044
Dialysis vintage	1.011 (0.988, 1.036)	0.351		
Systolic BP	1.027 (1.004, 1.051)	0.024	1.047 (1.007, 1.088)	0.020
Hemoglobin	0.960 (0.931, 0.990)	0.009	0.949 (0.907, 0.994)	0.026
Albumin	0.856 (0.757, 0.968)	0.013		
Phosphorus	3.255 (1.321, 8.021)	0.010		
GFR	1.017 (1.002, 1.033)	0.030	1.080 (1.038, 1.124)	< 0.001
Total Kt/V	0.079 (0.016, 0.388)	0.002	0.003 (0.001, 0.078)	0.001
NNmean	1.005 (1.001, 1.010)	0.023		
SDNN	0.955 (0.922, 0.989)	0.011		
ULF	0.997 (0.995, 0.999)	0.015		
VLF	0.998 (0.997, 0.999)	0.002		
LF	0.997 (0.995, 1.000)	0.019		
HF	0.998 (0.996, 1.000)	0.076		
TP	0.999 (0.999, 1.000)	0.002		
DC	0.765 (0.607, 0.963)	0.023		
SD2	0.955 (0.926, 0.984)	0.003		
ApEn	21.752 (0.602, 786.063)	0.092		
aSKNA	0.044 (0.006, 0.325)	0.002	0.056 (0.004, 0.797)	0.033

OR, odds ratio; CI, confidence interval; BP, blood pressure; GFR, glomerular filtration rate; ANS, autonomic activity system; NNmean, mean interval of normal sinus beats; SDNN, standard deviation of normal-to-normal R-R intervals; ULF, ultral low frequency power; VLF, very low frequency power; LF, low frequency power; HF, high frequency power; TP, total power; DC, deceleration capacity; ApEn, approximate entropy; aSKNA, average voltage of 5-min skin sympathetic nerve activity.

Data are presented as mean ± SD, numbers and percentages.

After inputting clinical and ANS indicators derived from univariate analysis and basic factors including age, sex and dialysis vintage into the multivariate Logistic regression model, we discovered that female sex (OR = 7.468, 95% CI: 1.054–52.942, *p* = 0.044), systolic BP (OR = 1.047, 95% CI: 1.007–1.088, *p* = 0.020), hemoglobin (OR = 0.949, 95% CI: 0.907–0.994, *p* = 0.026), GFR (OR = 1.080, 95% CI: 1.038–1.124, *p* < 0.001), total Kt/V (OR = 0.003, 95% CI: 0.001–0.078, *p* = 0.001), and aSKNA (OR = 0.056, 95% CI: 0.004–0.797, *p* = 0.033) were identified as independent predictors for RRF decline (Table 3).

### Correlation between ANS activity indexes and volume status

**Figure 3:**
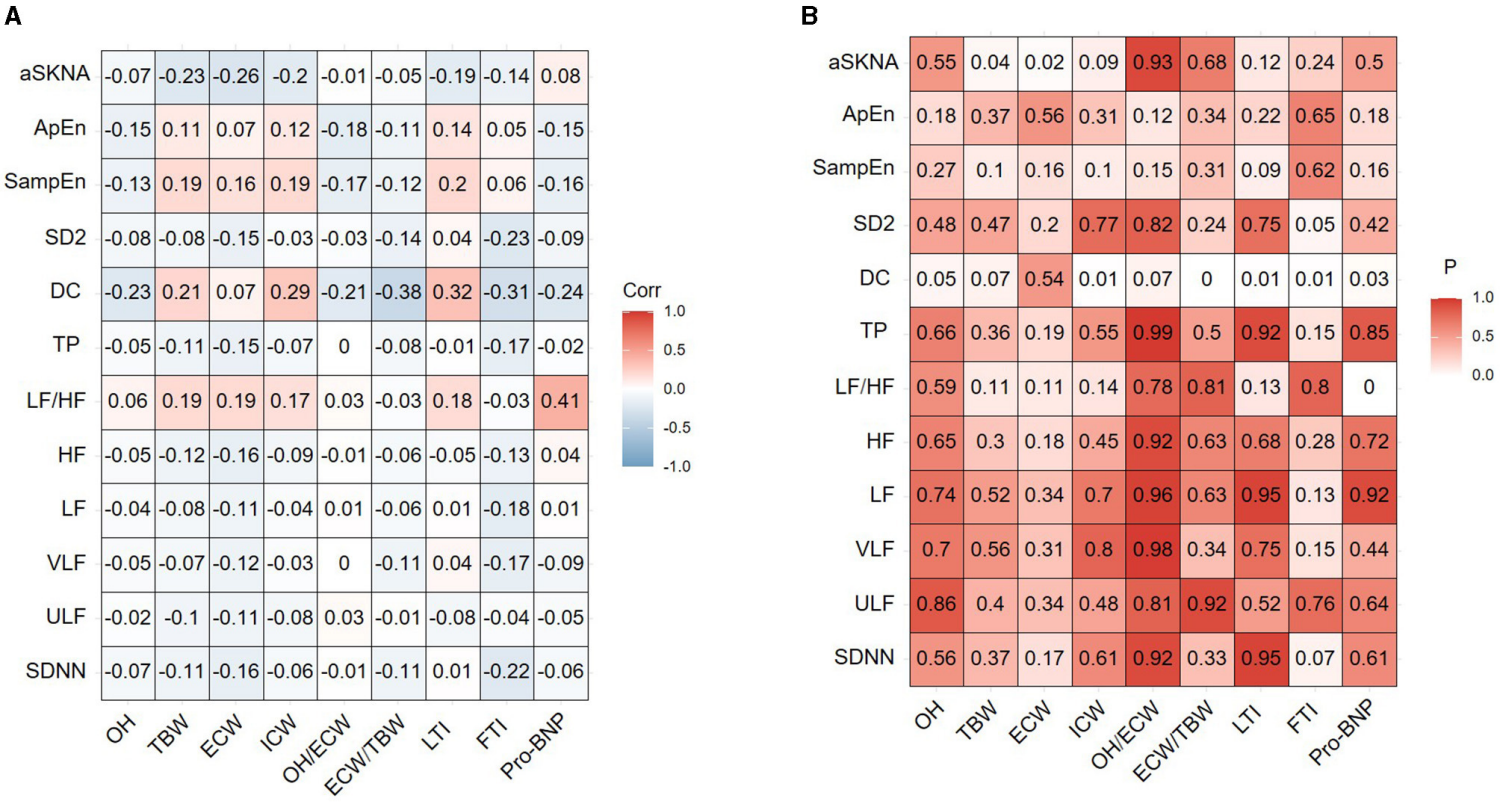
The correlation between ANS indexes and volume status indexes of the study population. **(A)** Correlation coefficient. **(B)** *P*-value. SDNN, standard deviation of normal-to-normal R-R intervals; ULF, ultral low frequency power; VLF, very low frequency power; LF, low frequency power; HF, high frequency power; LF/HF, ratio of LF power to HF power; TP, total power; DC, deceleration capacity; SampEn, sample entropy; ApEn, approximate entropy; aSKNA, average voltage of 5-min skin sympathetic nerve activity; OH, overhydration; TBW, total body water; ECW, extracellular water; ICW, intracellular water; LTI, lean tissue index; FTI, fat tissue index; pro-BNP, pro-brain natriuretic peptide.

**Figure 4:**
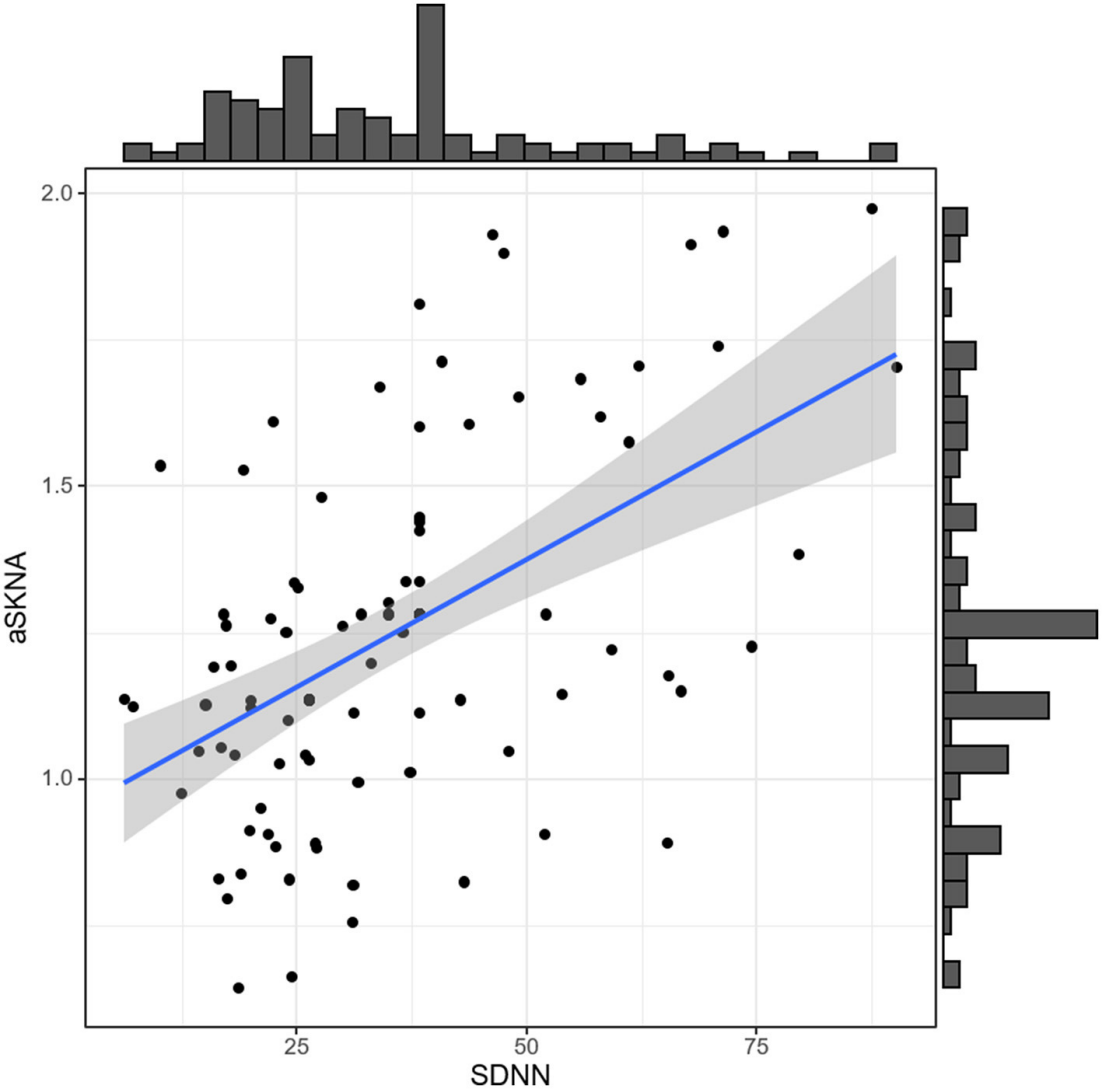
Spearman's correlation between SDNN and aSKNA in CAPD patients. Spearman correlation coefficient = 0.5; *p* = 8.118e-08. SDNN, standard deviation of normal-to-normal R-R intervals; aSKNA, average voltage of 5-min skin sympathetic nerve activity; CAPD, continuous ambulatory peritoneal dialysis.

We investigated the correlation between ANS activity indexes and volume status indicators (OH, ECW, ICW, TBW, OH/ECW, ECW/TBW, and pro-BNP) in the CAPD patients, respectively (Figure 3). It can be observed that there was a correlation between aSKNA and TBW (*r* = −0.233, *p* = 0.043), aSKNA and ECW (*r* = −0.262, *p* = 0.022), DC and ECW/TBW (*r* = −0.377, *p* = 0.0008), DC and pro-BNP (*r* = −0.238, *p* = 0.0033), respectively. Spearman's correlation analysis demonstrated that aSKNA and SDNN were moderately correlated (*r* = 0.5, *p* < 0.001; Figure 4). The correlation between ANS activity indexes and other blood parameters was shown in the Supplementary Figure 1.

### Nomogram model for predicting 1-year RRF decline in CAPD patients

**Figure 5:**
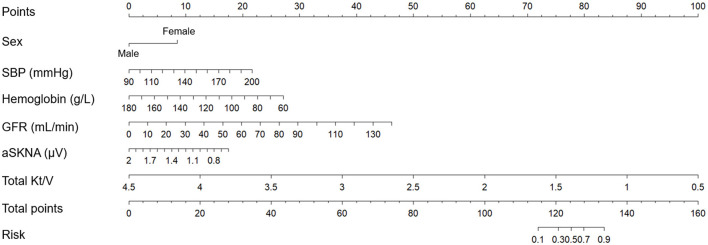
Nomogram model based on clinical factors and ANS indexes to predict 1-year RRF decline in CAPD patients. ANS, autonomic nervous system; RRF, residual renal function; CAPD, continuous ambulatory peritoneal dialysis; SBP, systolic blood pressure; GFR, glomerular filtration rate; aSKNA, average voltage of 5-min skin sympathetic nerve activity.

Nomogram model was used to establish models. Firstly, univariate analysis was used to examine the influence of baseline data and HRV indices on RRF decline. The results of the univariate and multivariate analysis were presented in Table 3. Among these characteristics, variables with p < 0.05 in the multivariate analysis, including female sex, systolic BP, hemoglobin, GFR, total Kt/V and aSKNA were integrated into the nomogram model (Figure 5).

**Figure 6:**
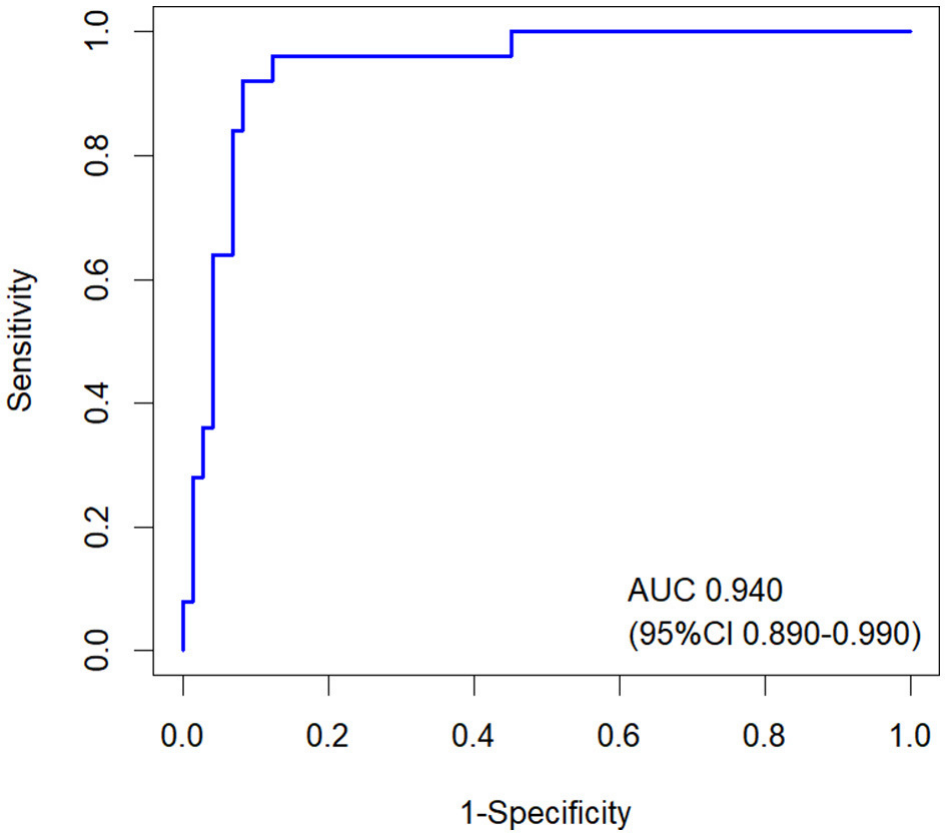
ROC curves of the nomogram model for predicting 1-year RRF decline in CAPD patients. The AUC of the nomogram was 0.940 (95% CI: 0.890–0.990). RRF, residual renal function; CAPD, continuous ambulatory peritoneal dialysis.

The nomogram model based on clinical factors (sex, systolic BP, hemoglobin, GFR, and total Kt/V) and aSKNA showed the largest AUC of 0.940 (95% CI: 0.890–0.990; Figure 6).

## Discussion

The main findings of this study are as follows: 1. RRF-D group generally exhibited lower HRV parameters and aSKNA compared with the RRF-S group. 2. a nomogram model based on clinical factors (sex, systolic BP, hemoglobin, GFR and total Kt/V) and aSKNA for predicting 1-year RRF decline in CAPD patients showed the largest AUC of 0.940 (95% CI: 0.890–0.990).

PD is a relatively useful and simple therapy strategy for kidney replacement. This continuous and gentle solute and fluid removal may preserve remaining nephrons and in turn maintain RRF (Auguste and Bargman, 2023). RRF has been shown to have a positive impact on PD patients in terms of reduced mortality (Bargman et al., 2001). The potential benefits of higher RRF include better volume control, reduced inflammation, improved nutritional status, reduced left ventricular hypertrophy, and lower levels of serum phosphate and uric acid (Tanriover et al., 2022; Alrowiyti and Bargman, 2023).

In ESKD patients, cardiovascular events are the primary cause of illness and death (de Jager et al., 2009), partly due to ANS dysfunction, which is mainly due to sympathetic hyperactivity and/or reduced parasympathetic activity (Salman, 2015). The analysis of HRV is a non-invasive method that can be used to assess cardiac ANS function. Reduced HRV is a significant predictor of symptoms and mortality in a wide range of diseases, especially in cardiovascular diseases (Sessa et al., 2018). In one study, the status of RRF in CAPD patients was positively correlated with LF/HF and negatively correlated with other HRV parameters including SDNN, SDSD, RMSSD, pNN50, LF, HF and TP (Tang et al., 2012). Similarly, we found that HRV parameters were generally lower in the RRF-D group compared with the RRF-S group, except LF/HF.

SKNA offers a new perspective for evaluating sympathetic nerve activity with second-by-second temporal resolution, which is not available with HRV (Kusayama et al., 2020). Our study demonstrated that SKNA value was an independent risk factor for RRF decline in CAPD patients. Furthermore, we initially identified a negative linear relationship between SKNA and bioelectrical impedance indexes including TBW and ECW.

To our knowledge, this is the first study to demonstrate a nomogram model based on ANS activity indexes to predict RRF decline in CAPD patients. We developed a simple and easy-to-use prognostic model integrating clinical parameters and ANS activity indexes for CAPD patients and the nomogram model might be a promising method for evaluating 1-year RRF decline.

### Limitation

This study has several limitations. Firstly, this research was conducted in a single center, and the sample size of patients was small, so the data may be biased. Secondly, the complexity of ANS makes it rather difficult to assess its function based on any isolated test. Thirdly, physical data were collected only once for each patient, and no long-term follow-up was formed, so there may be some contingency in the results. Finally, although clinical factors combined with ANS activity indexes achieved favorable predictive value in this study, the specific physiological mechanism of these factors is still unclear, and the underlying relationship between these factors and RRF decline needs to be further investigated.

## Conclusion

The nomogram model based on clinical factors (sex, systolic BP, hemoglobin, GFR, and total Kt/V) and ANS activity index (aSKNA) might be a promising method for predicting 1-year RRF decline in CAPD patients.

## Data Availability

The raw data supporting the conclusions of this article will be made available by the authors, without undue reservation.
