# Supplementary material for: Nomogram model based on clinical factors and autonomic nervous system activity for predicting residual renal function decline in patients undergoing peritoneal dialysis
Source: Front Neurosci. 2024 Nov 1;18:1429949. doi: 10.3389/fnins.2024.1429949 (PMC11564157; doi:10.3389/fnins.2024.1429949)
Supplement: Supplementary file 1 [file Data_Sheet_1.docx]

**Supplementary Figure 1.** The correlation between the ANS indexes and clinical characteristics of the study population.


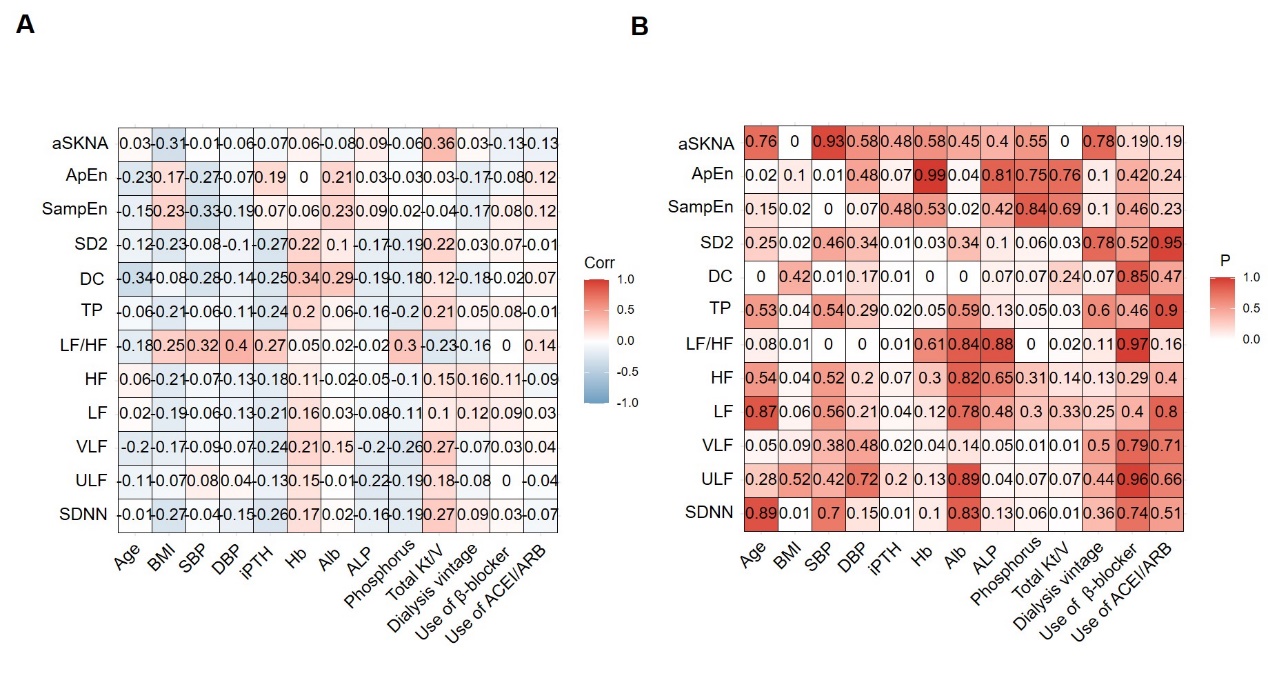


(A) Correlation coefficient. (B) P value. SDNN, standarddeviation of normal-to-normal R-R intervals; ULF, ultral low frequency power; VLF, very low frequency power; LF, low frequency power; HF, high frequency power; LF/HF, ratio of LF power to HF power; TP, total power; DC, deceleration capacity; SampEn, sample entropy; ApEn, approximate entropy; aSKNA, average voltage of 5-min skin sympathetic nerve activity; BMI, body mass index; SBP, systolic blood pressure; DBP, diastolic blood pressure; iPTH, intact parathyroid hormone; Hb, hemoglobin; Alb, albumin; ALP, alkaline phosphatase; ACEI/ARB, angiotensin-converting enzyme inhibitors/angiotensin receptor blocker.
